# High-Toughness Silk Produced by a Transgenic Silkworm Expressing Spider (*Araneus ventricosus*) Dragline Silk Protein

**DOI:** 10.1371/journal.pone.0105325

**Published:** 2014-08-27

**Authors:** Yoshihiko Kuwana, Hideki Sezutsu, Ken-ichi Nakajima, Yasushi Tamada, Katsura Kojima

**Affiliations:** 1 Silk Materials Research Unit, National Institute of Agrobiological Sciences, Tsukuba, Ibaraki, Japan; 2 Transgenic Silkworm Research Unit, National Institute of Agrobiological Sciences, Tsukuba, Ibaraki, Japan; 3 Faculty of Textile Science and Technology, Shinshu University, Ueda, Nagano, Japan; Institute of Farm Animal Genetics, Germany

## Abstract

Spider dragline silk is a natural fiber that has excellent tensile properties; however, it is difficult to produce artificially as a long, strong fiber. Here, the spider (*Araneus ventricosus*) dragline protein gene was cloned and a transgenic silkworm was generated, that expressed the fusion protein of the fibroin heavy chain and spider dragline protein in cocoon silk. The spider silk protein content ranged from 0.37 to 0.61% w/w (1.4–2.4 mol%) native silkworm fibroin. Using a good silk-producing strain, C515, as the transgenic silkworm can make the raw silk from its cocoons for the first time. The tensile characteristics (toughness) of the raw silk improved by 53% after the introduction of spider dragline silk protein; the improvement depended on the quantity of the expressed spider dragline protein. To demonstrate the commercial feasibility for machine reeling, weaving, and sewing, we used the transgenic spider silk to weave a vest and scarf; this was the first application of spider silk fibers from transgenic silkworms.

## Introduction

Orb-web spiders, such as *Nephila* and *Araneus* genera, produce a variety of silks that have excellent mechanical properties. For example, their dragline silks are among the strongest fibers, approximately threefold tougher than aramid fibers and fivefold stronger than steel [1].

The sequences of spider silk have been investigated extensively. In the case of dragline silk, partial cDNA sequences have been obtained from the major ampullate silk protein (MaSp) of *Nephila clavipes* (MaSp-1[2] and -2[3]) and *Araneus diadematus* (ADF-3[4] and -4[4]). There is a clear relationship between the sequences of these proteins and their structural properties [5]. Generally speaking, spider silk protein has a highly repetitive sequence. Within each repeat motif of *Nephila clavipes* and *Araneus diadematus*, a short polyalanine block forms a very short crystal domain. The remainder contains a glycine-rich amorphous domain. The network structure of the dragline contains a low volume fraction of small crystals, separated by long amorphous domains. This complex structure is thought to be the origin of the incredible strength, toughness, and viscoelasticity of spider dragline silks [5].

Numerous studies have investigated the production of artificial spider silks using *Escherichia coli*, [6]–[12] yeasts [13], mammalian cells [1], insect cells [14]–[16], plants [17], [18], animals [19], and silkworms [20]–[23]. However, long fibers can be obtained only from silkworms. Spider silk protein is highly repetitive and large; *Escherichia coli* and cultured cells are not suitable for the production of such proteins.

The silkworm, *Bombyx mori*, is able to efficiently spin proteins that consist of repetitive sequences [24]. Its silk is composed of fibrous fibroin contained within a sericin protective coating. The fibroin consists of three proteins: fibroin heavy chain (H-chain), fibroin light chain (L-chain), and fibrohexamerin protein (fhx/P25). The H-chain is believed to be associated with the mechanical properties of silk. A transgenic technique for the silkworm was established in 2000 [25]. In 2007, we reported fibroin H-chain modification [26] and the development of transgenic silkworms that expressed spider (*Araneus ventricosus*) dragline proteins [20]. In the 2007 report, a 2.5-kbp native cDNA sequence, the longest partial cDNA sequence of *Araneus ventricosus* dragline silk protein among those reported previously, was expressed in the silk fibroin of the w1-pnd silkworm, an experimental silkworm strain. Because the w1-pnd silkworm strain produces only poor cocoons, having a non-uniform thickness and ill-formed silk-fiber shape, we encountered great difficulty in measuring the physical properties of the silk. As a result, only the structural analysis of the silk was performed using nuclear magnetic resonance (NMR); results indicated that the silk structure was not significantly changed by incorporation of spider dragline silk protein.

In previous research on silkworms [20]–[23], the tensile properties were measured using a single transgenic silk fiber from a cocoon. For industrial usage of the transgenic silk, the raw silk, that is a machinery-produced bundle of silk threads, is needed. The w1-pnd strain or other experimental silkworm strains were used in previous studies. However, the limitations presented by the poor physical properties of these silks prevented precise measurement of their tensile properties; as a result, reeling of these raw silks from cocoons and textile weaving with these silks could not be performed. Although the experimental strains are useful for establishing the transgenic strains, they are not suitable for producing silks that can be applied in practice. Therefore, to analyze the tensile properties of the transgenic silk fiber and to use the transgenic silk in practical applications, the use of a silkworm strain that can produce smooth, uniform silk, such as C515, was necessary. Additionally, the use of the C515 strain allowed closer examination of the tensile properties of raw silk produced from transgenic cocoons for the first time.

To analyze more precisely the physical properties of transgenic silk, herein we generated transgenic silkworms using the C515 strain, which expresses the modified spider dragline protein reported previously [20]. Further, unlike previous studies [20]–[23], we focused on raw silk, which is more important for industrial technologies, in addition to cocoon silk, with the goal of using transgenic spider silk in commercial textiles. The toughness of the transgenic raw silk was 53.2% higher than that of native silk. We also demonstrated the use of transgenic silk as a new high-performance biopolymer by reeling and weaving the silk, and sewing the resultant silk fabric with an industrial machine.

## Materials and Methods

### cDNA library

A cDNA library from the silk glands of the garden spider, *Araneus ventricosus*, was obtained as follows. *Araneus ventricosus* individuals were collected in a field at our institute in Tsukuba, Ibaraki, Japan, and all silk glands were harvested. Then, poly(A)+ RNAs were extracted from each silk gland, followed by cDNA synthesis with oligo-dT primers. Then cDNA libraries were created using the directional cloning method (Lambda Zap cDNA cloning kit, Stratagene).

The cDNA clones prepared from the *Araneus ventricosus* ampullaceal gland were picked randomly. Sequences of both the 5′ and 3′ ends of each cDNA were determined using a DNA sequencer (ABI3730XL, Applied Biosystems). The sequences were compared with known spider dragline silk genes and were found to be a match with that of *Araneus ventricosus*. The obtained sequences were analyzed via step-by-step sequencing using specific primers. The longest sequence in the dragline silk genes, designated “SpA”, was used in the following analysis.

### Construction of plasmid

A modified fibroin H-chain construct was generated as follows. First, plasmid pHC-enhanced green fluorescent protein (EGFP) [27] was improved by adding a 6x-His-tag at the C-terminus by polymerase chain reaction (PCR). The C-terminal and poly(A) regions of Fib-H were amplified with primer sets (for the C-terminal, CTD-5: 5′-GGGGTCGACA GCGTCAGTTA CGGAGCTGG-3′ and CTD-3His6: 5′-GGGACGCGTT TAGTGGTGAT GGTGGTGATG GCAATTCACA CAAGG-3′; and for the poly(A) region, poly(A)-5: 5′-GGGACGCGTT TTTTAATATA AAATAACCC-3′ and M13 reverse primer) using pHC-EGFP as a template. Then, the corresponding sequence in pHC-EGFP was replaced with the amplified sequences. The resultant plasmid was designated pHChis6-EGFP. Finally, a multi-cloning site (*Bam* HI–*Cla* I–*Pac* I–*Xba* I–*Sal* I) was introduced to replace the EGFP sequence, using a primer set (MCS-5: 5′-GATCCATCGA TTTAATTAAG TCTAGAG-3′ and MCS-3: 5′-TCGACTCTAG ACTTAATTAA ATCGATG-3′) and designated pHChis6-mcs.

The cloned spider dragline gene was cut with *Sma*I and *Xba*I and subcloned between *Xba* I and blunt-ended *Bam* HI sites of pHChis6-mcs; the resultant plasmid was designated pHChis6-SpA. Finally, pHChis6-SpA and pHChis6-EGFP were digested with *Asc*I and *Fse*I, respectively, and cloned into the *Asc* I–*Fse* I region of pBac[3XP3-DsRed2afm]_HC-EGFP to generate pBac[3XP3-DsRed2afm]_HChis6-SpA and pBac[3XP3-DsRed2afm]_HChis6-EGFP, respectively, as shown in Figure S1.

### Production of a transgenic silkworm

**Figure 1:**
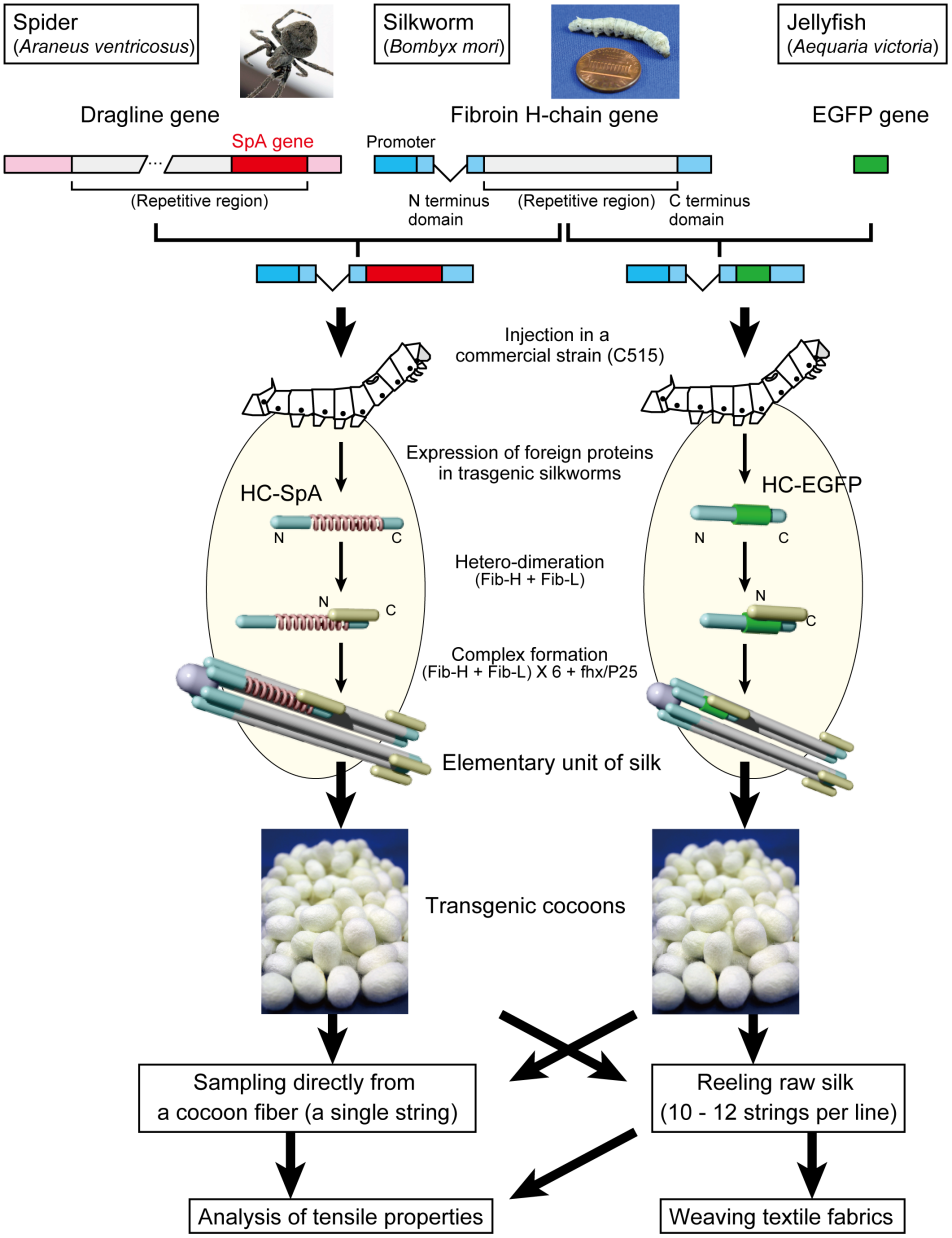
Overview of the strategy used in this study. We cloned a partial sequence spider (*Araneus ventricosus*) dragline silk. The spider dragline protein (SpA) gene or enhanced green fluorescent protein (EGFP) gene were fused between the N- and C-terminal domains of the fibroin H-chain protein gene and the transgenic silkworm expressing these modified proteins, respectively. The transgenic silkworms expressed spider dragline protein or EGFP as a part of the silk fibroin proteins. The elementary units, consisting of fib-H, fib-L, and fhx/P25, were secreted into the lumen of the silk gland, and the silk was spun into a cocoon. The single cocoon silk and reeled raw silk were then prepared and their tensile properties analyzed. The raw silk was woven into yarn by an industrial machine to demonstrate its suitability for commercial applications.

C515, a Japanese silkworm strain that produces fine silk fiber, was used as the host for a transgenic silkworm. Silkworm transgenesis was performed as described in previous research [25] with minor modifications. Silkworm eggs were acid-treated for 30 min at 3 h post-oviposition. The DNAs of the plasmids, pBac[3XP3-DsRed2afm]_HChis6-SpA or pBac[3XP3-DsRed2afm]_HChis6-EGFP, were then injected into the eggs, along with a helper plasmid, pA3help, at 6 to 10 h post-oviposition. Hatched larvae (G0) were reared and permitted to mate with each other or with C515. Six to seven days after oviposition, the resultant embryos (G1) were screened for transgenic individuals with DsRed2 expression, using a fluorescence microscope (MZ16 FA, Leica). The transgenic silkworms were reared together and sib-mated for 16 generations with sequential screening by strong excitation of DsRed2 fluorescence in the eyes; quantitative analysis was performed to determine the number of transgenes. The resultant strains were designated C515-SpA1 and C515-SpA2 (from pBac[3XP3-DsRed2afm]_HChis6-SpA injection) and C515-EGFP (from pBac[3XP3-DsRed2afm]_HChis6-EGFP injection). In additional experiments, an inbred strain that was a mix of C515-SpA1 and -SpA2 was also established, and designated as C515-SpA1×2. Transgene analysis was performed on the sixteenth generation, and the silks obtained from the next generation were used for protein analysis and tensile tests. In this paper, the recombinant proteins expressed from SpA and EGFP sequences, with a fibroin H-chain, are called “HC-SpA” and “HC-EGFP”, respectively, as shown in Figure 1 and Figure S1.

### Southern hybridization

Probe DNA was prepared as described in our previous report [26]. Genomic DNAs of wild-type (C515), C515-SpA1, C515-SpA2, and C515-EGFP silkworms were prepared from adult moths as follows. First, the adult body was homogenized in 2 mL of homogenization buffer (50 mM Tris-HCl (pH 8.0), 100 mM NaCl, 10 mM EDTA, 1% SDS and 0.2 mg mL^–1^ proteinase K); the mixtures were shaken for 1 h at 50°C. After being shaken, the homogenates were extracted once with TE-saturated phenol, once with TE-saturated phenol–chloroform–isoamyl alcohol, and then precipitated with ethanol.

About 5 µg of each genomic DNA were digested with *Kpn*I or *Hind*III. Digested DNA (2 µg) was applied to 0.8% agarose gel electrophoresis and then blotted onto a Hybond N+ membrane (GE Healthcare). The *piggyBac* R-arm sequence was then detected with an AlkPhos Direct Labeling and Detection Kit (GE Healthcare), using the PCR-amplified *piggyBac* R-arm sequence [26], in accordance with the manufacturer's instructions. Signals were detected with an LAS-3000mini compact chemiluminescence system (Fujifilm Corporation).

### SDS-PAGE and Western blot

Three or four cocoons for each silkworm strain were degummed by 8 M urea with 0.5% 2-mercaptoethanol (Wako Pure Chemical Industries). The resultant fibroins were dissolved in a 9-M lithium bromide aqueous solution, dialyzed against deionized water, and then adjusted to 10 mg mL^−1^ with deionized water. Sodium dodecyl sulfate-polyacrylamide gel electrophoresis (SDS-PAGE) was performed on the fibroins, using Mini-PROTEAN TGX Precast Gels (Any kD, Bio-Rad Laboratories). Western blotting was used to confirm the expression of the spider silk protein. Proteins were separated by SDS-PAGE, blotted onto a PVDF membrane (Immobilon-P, Millipore), and subjected to Western blotting with ECL-Plus (GE Healthcare), in accordance with the manufacturer's instructions. Signals were detected with an LAS-3000 mini compact chemiluminescence system. Anti-6xHis-tag antiserum (courtesy of Dr. Mitsuru Sato, NIAS) and anti-SpA-specific peptide antibody (Operon Biotechnologies) were used as the primary antibodies. The anti-SpA-specific peptide antibody used here was raised in a rabbit, against the peptide “TGGRAGGPKAGPG”, which corresponds to the garden spider (*Araneus ventricosus*) dragline silk protein; it was then affinity-purified with the target peptide and subjected to Western blot assay.

To identify the composition of the recombinant spider silk protein, HC-SpA, in silk, a relative amount of HC-SpA was quantified against that of the fibroin-L chain protein, as follows. Fibroin proteins were prepared from the cocoons as mentioned earlier. The silk solutions were then dialyzed overnight against 4 M urea. The silk proteins were separated by SDS-PAGE and stained using a fluorescent dye (Oriole fluorescent gel stain, Bio-Rad Laboratories). Finally, the amount of proteins was digitized using an ultraviolet (UV) illuminator (Printgraph, ATTO) and data acquisition software (E-shoot, ATTO). The digitized data were then analyzed by densitometry (CS Analyzer, ATTO).

### Tensile analysis of transgenic silk

#### Preparation of transgenic silk

To reel a single silk fiber from a cocoon, each cocoon was first cooked in deionized water for 1 min at 95°C. The cooked cocoon was then soaked in cold water for 1 min. This process was repeated twice. The sericin layer swelled during this process, thereby assisting reeling a fiber from the cocoon. After the cocoon swelling process, a single silk fiber was reeled at 100 mm s^−1^ using a rolling drum; the drum was precisely controlled with a pulse motor (PMCD-05, Tsujicon), using as little excess tension as possible.

A sampling sheet was prepared, as shown in Figure S2 A. A reeled fiber from the middle part of the cocoon was attached to the sampling sheet over the holes by adhesive tape. The diameters of the attached fibers were measured with a digital microscope (VH-8000, Keyence). In this paper, the diameter is defined as shown in Figure S2 B. We calculated the cross-sectional area using this diameter, under the assumption of an elliptical cross-sectional shape for the cocoon silk fiber. Because the measured diameters included the thin sericin layer, the diameters and the cross-sectional areas were consistently overestimated, compared with a real silk fiber, which lacks the sericin layer. As a result, the values for the tensile properties, such as the breaking stress, Young's modulus, and toughness, may have been underestimated relative to a real silk fiber.

Raw silk from C515 and transgenic cocoons was prepared according to Japanese-authorized cocoon testing methods, as follows. Cocoons were cooked in an automatic cooking machine (VP type, Harada) at 50–95°C for 15 min. After this heat treatment, 27-denier raw silk was reeled, using an automatic reeling machine (CT-2, Nissan) at a speed of 150 m min^−1^. The reeled raw silks were used in tensile tests and fabric making.

#### Tensile tests

**Figure 2:**
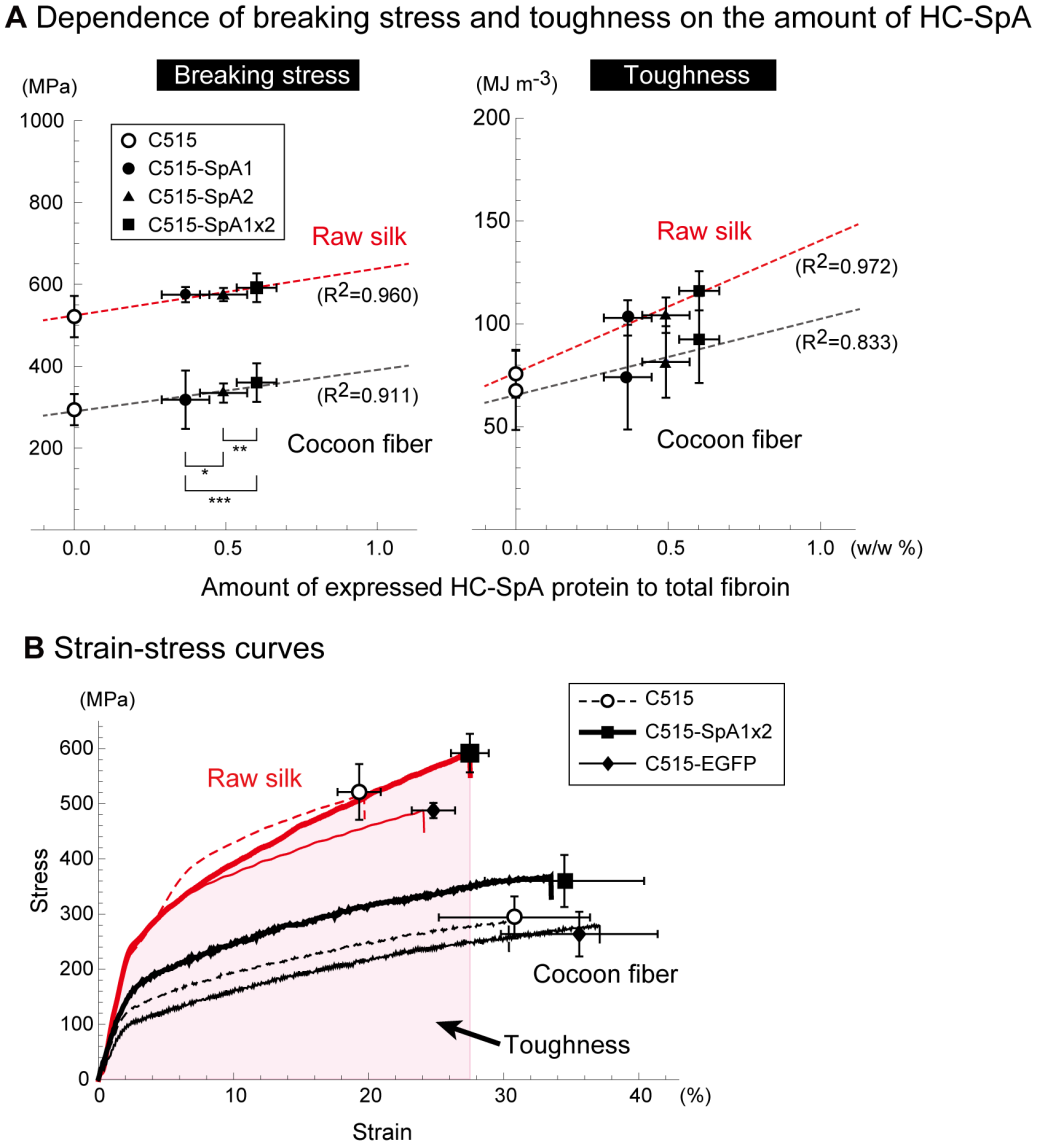
Dose dependence of tensile properties in C515-SpA silks (A) and representative stress–strain curves (B). (A) Breaking stress (left) and toughness (right) are plotted as a function of the amount of HC-SpA protein (%) in total fibroin proteins. The amounts of HC-SpA proteins in total fibroin proteins, estimated by SDS-PAGE using densitometry, were 0.37 (±0.08 s.d.), 0.49 (±0.08 s.d.), and 0.61% (±0.07 s.d.) w/w in C515-SpA1, C515-SpA2, and C515-SpA1×2, respectively (n = 21). The p-values of a one-tailed *t*-test indicated significant differences in the amounts of each transgenic strain (p-values: * 3.1×10^−6^, ** 7.8×10^−6^, *** 2.4×10^−13^). The broken lines are the regression lines obtained by the least-squares method. The breaking stress and toughness were high for raw silk; the highest values were found for the C515-SpA1×2 silks, which contained the most HC-SpA protein. The breaking stress and toughness were proportional to the HC-SpA content for both cocoon fiber and raw silk. (B) Typical strain–stress curves with average breaking points are shown for each strain. Circles, rectangles, and diamonds show the average strain and stress values. Error bars correspond to the standard deviations. All values are shown in Tables S1–S3. There were clear differences between the cocoon fibers and the raw silks; cocoon fibers were more elastic with low breaking stress, while raw silks had low elasticity and high breaking stress. For the raw silks, the C515-EGFP silks showed high elongation and low breaking stress, compared with those of C515. C515-SpA1×2 silks showed the highest breaking stresses, elongation, and toughness (shaded area).

Single-fiber mechanical testing was performed under ambient conditions (25–28°C, 45–50% relative humidity) using an EZ Test (Shimadzu) instrument, equipped with a 5-N load cell. For raw silks, another instrument (Tensilon RTA-100, Orientec), having a 5-kgf load cell, was used at 20°C, 65% relative humidity. In both experiments, specimens were equilibrated under the indicated conditions for 24 h, prior to tensile testing. For cocoon fibers, the specimen length was 20 mm; the measured data were recorded using control software (Trapezium2, Shimadzu) at a strain rate of 10 mm min^−1^ and a sampling rate of 10 Hz. For raw silk, the specimen length was 10 cm; the measured data were recorded at a strain rate 150 mm min^−1^ and a sampling rate of 375 Hz. The toughness values were calculated from stress–strain curves, by integrating the hatched area shown in Figure 2 B with mathematical software (Excel, Microsoft; Mathematica, Wolfram Research).

Tensile properties were measured for over 40 single fibers in each experiment. Statistical analysis was performed using the F-test and Student's t-test using the Excel or Mathematica software. First, an F-test was performed to determine if there was a significant difference between variances. Then, Student's *t*-test was performed to determine if the averages were significantly different. Differences were considered significant when p-values were less than 0.01.

### Weaving of a textile using transgenic silks

To investigate the possibilities of transgenic silk weaving, we made a vest and a scarf using transgenic silk from the cocoons of C515-SpA2. Eight pieces of 27-denier raw silk were plied. The twisted yarn was then degummed with a 0.1% alkaline enzyme for 1 h at 60°C. The degummed yarn was dyed by acid dyes (Kayanol Milling Blue GW and Inolar Fast Red MNW) for 20 min at room temperature. The dye temperature was then raised to 90°C over 60 min, and the temperature was held there for 90 min. Acetic acid (5%, based on the fiber weight) was also added to the dye, 10 min after the start of dyeing. At the end of the dyeing process, the yarn was washed with water and the degummed and dyed silks were knitted by a machine (SMS 330 TC4, Stoll Japan).

## Results and Discussion

### Overview of a transgenic silkworm expressing spider dragline protein

Figure 1 provides an overview of our transgenic silkworm, which expresses spider dragline silk protein, and EGFP as a part of the fibroin. First, the partial sequence of the spider (*Araneus ventricosus*) dragline silk protein gene, SpA, was cloned, and the repeat sequence of SpA (2149 nts) was then subcloned between the N- and C-terminal domains of the *Bombyx* fibroin H-chain gene [26]. After generating the transgenic silkworm bearing this modified fibroin H-chain gene with *piggyBac* vector system, the transgenic silkworm expressed the modified protein, HC-SpA, in the silk gland. The HC-SpA protein was then dimerized with the fibroin L-chain, and assembled into an elementary silk unit in the silk gland of the silkworm. Finally, the transgenic silk, which included the spider dragline protein, was spun into a cocoon. Silk fibers were collected as a single fiber and as raw silk from each cocoon and then analyzed. As a control, an EGFP-expressing transgenic silkworm, instead of SpA, was also generated and used.

### Cloning of spider dragline protein genes

We first created a cDNA library from the ampullate silk gland of the spider (*Araneus ventricosus*) and selected one cDNA that corresponded to the dragline silk gene. The cDNA selected corresponded to the longest sequence and was designated “SpA”, AB829892 (DDBJ); its sequence is shown in Figure S3. The SpA gene was 2513 bp long and included one partial open reading frame (ORF) of 803 amino acids and an 82-bp 3′ non-coding region, followed by an 18-nt poly(A) sequence. The ORF contained the highly repeated (18 copies) sequence of a polyalanine block, many “GPGXX” motifs apparent in ADF-3 [4], and a C-terminal non-repeated amino-acid sequence. Collectively, these features indicated that the cloned sequence had the typical characteristics of the 3′ terminal in spider dragline silk proteins, especially the *Araneus diadematus* fibroin 3 (ADF-3)-like protein [4].

### Transgenic silkworms

We generated transgenic silkworms using a Japanese commercial silkworm strain, C515. Generally, the w1-pnd experimental strain of silkworm has been used for transgenesis. Since the experimental strains were generated without concern for silk production, the physical properties of the silk fibers could not be properly analyzed. The phenotype of C515 is unsuitable for transgenesis, but its silk is ideal for fiber analysis. We generated three transgenic silkworm strains in this study using C515; the transgenic efficiencies were 16.7% (C515-SpA1), 22.6% (C515-SpA2), and 20.0% (C515-EGFP), which were almost identical to other transgenic silkworms generated to date (average of all germline transformation efficiencies: 15.0±10.7% (s.d.)). The transgenes in each strain were detected by Southern hybridization (Fig. S4), which identified three insertions in each transgenic line. These results indicate that the C515 strain is a good host for transgenesis, and, in fact, many silkworm strains without w1-pnd phenotypes could potentially host transgenesis. These transgenic silkworm strains were maintained for over 15 generations by sib-mating and sequential analysis of transgenes. We obtained one race for each transgenic strain. The three strains specified above and the F1 cross of C515-SpA1 and C515-SpA2 were used for further analysis.

**Figure 3:**
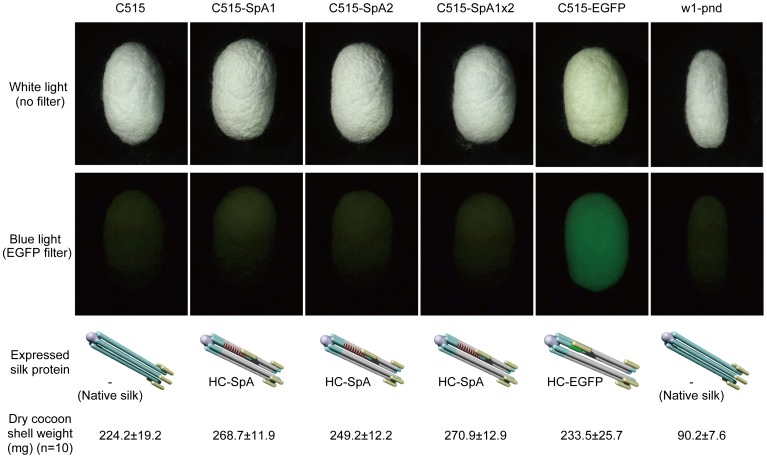
Comparison of the cocoons in a commercial (C515) and an experimental strain (w1-pnd). Schematic diagrams show the expressed silk elementary units and average cocoon shell weights of the C515 and transgenic cocoons, along with the w1-pnd cocoon, which is used widely as a host for the transgenic silkworm. The C515 and transgenic cocoons had comparable shapes and weights, while the w1-pnd cocoon was small and light. Raw silk could not be obtained from w1-pnd cocoons, because it was difficult to reel from the cocoons. The C515-EGFP cocoon showed green fluorescence, derived from the expressed modified protein HC-EGFP, indicating that the expressed modified protein formed its native tertiary structure. The HC-SpA protein expressed in the silks also formed its native structure, and contributed to high tensile properties.

Figure 3 shows the cocoons of transgenic and parental C515 silkworms, along with w1-pnd. A cocoon of the C515-EGFP transgenic silkworm showed green fluorescence under fluorescence microscopy, while the other transgenic silkworm cocoons did not. Green fluorescence of C515-EGFP cocoons indicated that the EGFP protein formed its natural “beta-barrel structure” in the spun silk fiber. Hence, the HC-SpA protein expressed in the silk was also expected to be expressed in the silk fibroin and form its natural structure in the cocoons. Additionally, the weights of the C515-SpAs and C515-EGFP cocoon shells were almost the same as that of the C515 shell, indicating that the expression of the fusion protein did not affect the silk productivity of the transgenic silkworms. On the other hand, the cocoon of w1-pnd was about one-third the weight of the C515 cocoon. This result clearly indicates that the C515 strain is more suitable for silk production and that the silk from the C515 strain was more suitable for precise tensile analysis.

### Spider silk protein expression in a transgenic cocoon

**Figure 4:**
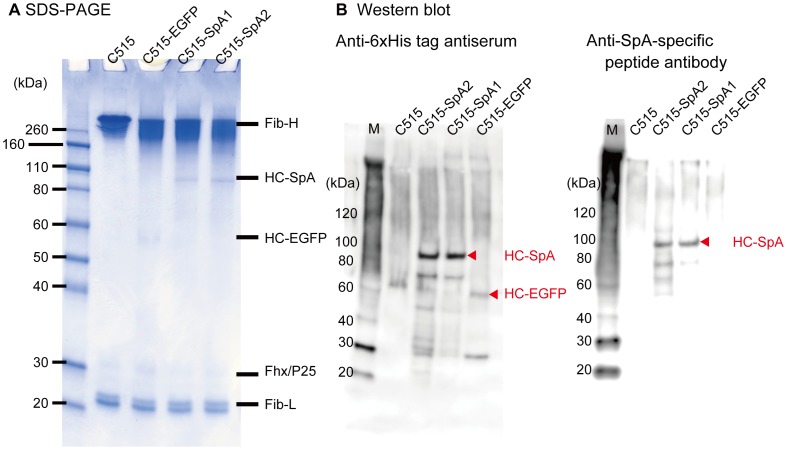
Sodium dodecyl sulfate-polyacrylamide gel electrophoresis (SDS-PAGE) and Western blotting assay of degummed silk proteins. (A) The fibroin proteins of the cocoons of C515 and three transgenic silkworms prepared in this study were analyzed. The cocoons were urea-degummed, and the resultant fibroin fibers were dissolved in 9-M aqueous lithium bromide solution, dialyzed against deionized water, and applied to SDS-PAGE. Fibroin H-chain protein, fibroin L-chain proteins, fhx/P25, and the fusion proteins, HC-SpA and HC-EGFP, were observed. (B) Western blotting analysis of fibroin proteins was performed using anti-6xHis tag antiserum (left) and anti-SpA protein specific peptide antibody (right). Fibroin proteins, such as C515, were not detected by these antibodies. HC-SpA protein, which contains 6xHis-tag and SpA proteins, could be detected with anti-6xHis tag antiserum and anti-SpA peptide antibodies. HC-EGFP protein, which contains EGFP and 6xHis tag, could only be detected using anti 6xHis-tag antiserum. Small amounts of degraded protein, which were not identified by SDS-PAGE, were present.

To identify the expression of HC-SpA and HC-EGFP proteins in fibroin, SDS-PAGE analysis and Western blot analysis were carried out using degummed fibroin proteins (Figure 4 A). All samples contained the fibroin L-chain protein (27 kDa), fhx/P25 protein (ca. 30 kDa), and fibroin H-chain protein (300 kDa). Additionally, specific proteins having a molecular weight around 100 kDa in were also present C515-SpA1 and C515-SpA2. These proteins were identified as the fusion protein HC-SpA, after detection by anti 6xHis-tag antiserum and anti-SpA-specific peptide antibodies (Figure 4 B). Also, the ca. 60-kDa protein detected in C515-EGFP silk was also identified as a fusion protein, HC-EGFP, by comparison with our previous work [20] and its detection using only anti 6xHis-tag antiserum (Figure 4 B).

Using densitometry, we calculated the amounts of HC-SpA proteins by comparison with the amount of fibroin L-chain protein. The composition of the fibroin L-chain is strictly regulated [28]. Thus, calibration curves for the amount of the fibroin L-chain, obtained using a series of sequentially diluted silk solutions, allowed the relative amount of HC-SpA protein to be calculated. Because the elementary silk unit consists of 6xH-chain, 6xL-chain, and 1xfhx/P25 proteins [28], the relative amount of L-chain in the total fibroin was calculated from the molecular weight; it was about 8.1% w/w of the total fibroin. The calculated amounts of HC-SpA in C515-SpA1, C515-SpA2, and C515-SpA1×2 against the total fibroin were 0.37 (±0. 08 s.d.), 0.49 (±0.08 s.d.), and 0.61% (±0.07 s.d.) w/w, respectively. In other words, the molar amounts relative to the fibroin H-chain were 1.4 (±0.3 s.d.), 1.9 (±0.3 s.d.), and 2.4 (±0.3 s.d.)%, respectively. These are significantly different according to the t-test (one-tailed); the p-values are shown in Figure 2 A. We also estimated the amount of HC-EGFP protein in C515-EGFP cocoon silk using a method reported previously [26]; it was about 1–2% w/w total fibroin according to SDS-PAGE analysis.

The difference in the HC-SpA protein expressed in C515-SpA1 and -SpA2 cocoons may result in the position effect of the transgene. The enhanced expression observed in the crossing strain, C515-SpA1×2, suggests a heterosis effect, or crossing of different strains. This phenomenon is applicable to mass production of the transgene with transgenic silkworms.

### Tensile properties of silk fibers from the transgenic silkworm

**Figure 5:**
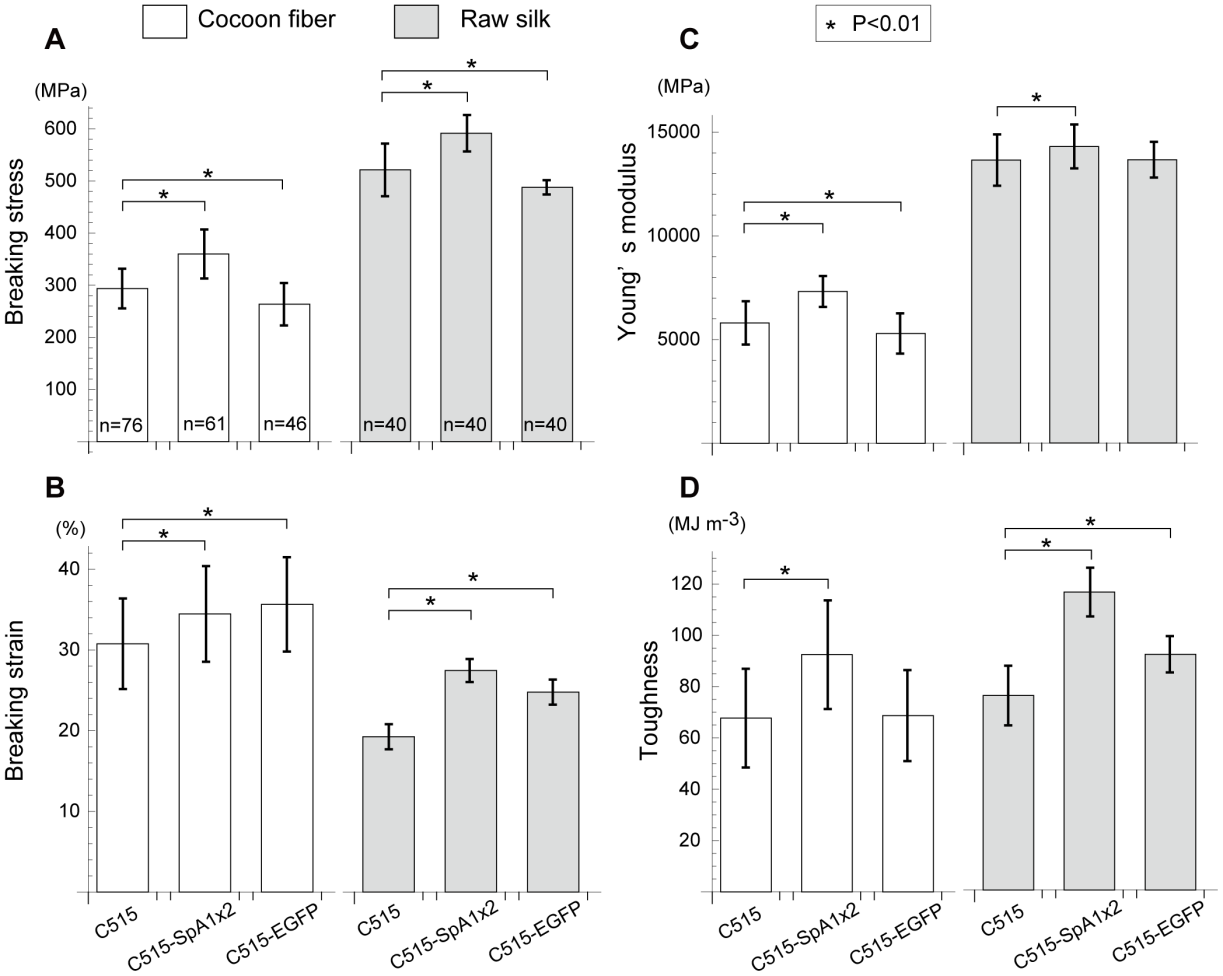
Tensile properties of the cocoon fiber and raw silk. The tensile properties are shown for cocoon silk (open box) and raw silk (filled box): breaking stress (A), breaking strain (B), Young's modulus (C), and toughness (D) for wild-type silk (C515), HC-SpA-expressed silk (C515-SpA1×2: F1 breed of C515-SpA1 and C515-SpA2), and HC-EGFP-expressed silk (C515-EGFP). The average values and their standard deviations (s.d.) are shown; detailed data and *p*-values are given in Tables S1–S3. For cocoon silk (open boxes), HC-SpA-containing cocoon silk (C515-SpA1×2) showed improvement in all properties, while C515-EGFP-containing silk showed only improved elongation. This indicated that expressed HC-SpA protein specifically improved the tensile properties of silk. This improvement was also evident, and more apparent, with raw silk as a consequence of the high speeds and high tension used in the reeling process. The breaking stress and breaking strain of C515-SpA1×2 raw silk improved by 13.5% and 42.5%, respectively, over that of C515 and resulted in an increase in toughness of 53.2%. In the HC-EGFP-containing raw silk, the breaking strain increased; however, the breaking stress decreased relative to C515. This means that the incorporation of HC-EGFP protein in silk weakens the raw silk and renders it more elastic. An asterisk “*” indicates a significant difference from C515 (i.e., *p*<0.01). The number of fiber specimens is shown in the breaking stress graph.

Thus far, the physical properties of transgenic silk with spider dragline silk have been analyzed using only a single fiber from a cocoon, because the silk was too weak to spin into raw silk [21]–[23]. In this study, using the C515 silkworm strain for transgenesis, raw silk could be pulled from the cocoons of transgenic silkworms and their physical properties analyzed for the first time. The raw silk was prepared as a bundle of about ten cocoon fibers, which were prepared via industrial processing. The relationship between the physical properties and the amount of HC-SpA protein in silk was plotted in terms of the breaking stress and toughness, and then compared with the properties of C515-SpA1, C515-SpA2, and C515-SpA1×2 silks (Figure 2 A and Table S1). All *t*-test p-values (one-tailed) are given in Tables S2 and S3. In both single-cocoon silk and raw silk, the breaking stress and toughness depended on the amount of HC-SpA protein, with significant improvements noted for the incorporation of HC-SpA protein at 0.4–0.6% w/w of the silk protein. The raw silk exhibited higher scores. Judging from the regression lines of single-cocoon silk and raw silk, if the HC-SpA content was raised to 5–8%, then the breaking stress of cocoon silk and raw silk would be equal to that of spider dragline silk (i.e., ∼1.1 GPa[5]). Among the three HC-SpA-expressing silks, C515-SpA1×2 silk, obtained by crossing C515-SpA1 and C515-SpA2, showed superior physical properties. The physical properties of C515-SpA1×2 were then compared with those of C515 and C515-EGFP (Figure 2 B). Clear differences were evident in the stress–strain curve between the cocoon fiber and raw silk. Our results indicated a sharp difference between single-cocoon silk and raw silk in the values for Young's modulus (also shown in Figure 5). This difference was attributed to the high speeds and excess tension applied during the reeling process from cocoon fiber to raw silk [29].

Former works on single cocoon silk with spider silk protein also showed improvements in the physical properties of the silk, in terms of strength, elongation, and Young's modulus [21]–[23]. In the case of a single cocoon fiber, our data agreed with previous results (Figure 5). In the case of raw silks, important in the production of silk fibers, Young's modulus for the different silk samples appeared to be fairly comparable; only differences in elongation (19.3% in C515, 27.5% in C515-SpA1×2, and 24.8% in C515-EGFP) appeared to have an effect on toughness (Figure 2 B). C515-SpA1×2 silk retained high elongation in raw silk, whereas the elongation of C515 and C515-EGFP decreased dramatically (Figure 2 B).

Figure 5 shows the precise tensile properties of breaking stress (A), breaking strain (B), Young's modulus (C), and toughness (D) on the cocoon fiber and raw silk of C515, C515-EGFP, and C515-SpA1×2 (which contains the highest amount of HC-SpA protein). All measured values are listed in Table S1. The results clearly showed that single cocoon fiber and raw silk have different tensile properties, as shown in Figures 2 and 5.

For all of the tensile properties evaluated in this study, C515-SpA1×2 cocoon silk had the highest scores for cocoon fibers. It had a superior breaking strain (22.5%), breaking stress (12.0%), Young's modulus (26.1%), and toughness (36.6%), compared with the native silk, C515. The improvement was even more evident when compared with raw silk, especially for toughness (116.1 MJ m^−3^ and 53.2% better than that of C515). C515-SpA1×2 exhibited better toughness, compared with that of synthetic rubber (100 MJ m^−3^) and the dragline of spider (*Nephila clavipes*, 111 MJ m^−3^) [30]. While the C515-EGFP silks showed only a moderately lower improvement in properties (or an improvement almost identical to that of C515), the C515-EGFP silk appeared to be weak and more elastic. These results indicated that the physical properties of the silk changed as a result of the characteristics of the incorporated proteins.

### Weaving using C515-SpA2 silk

**Figure 6:**
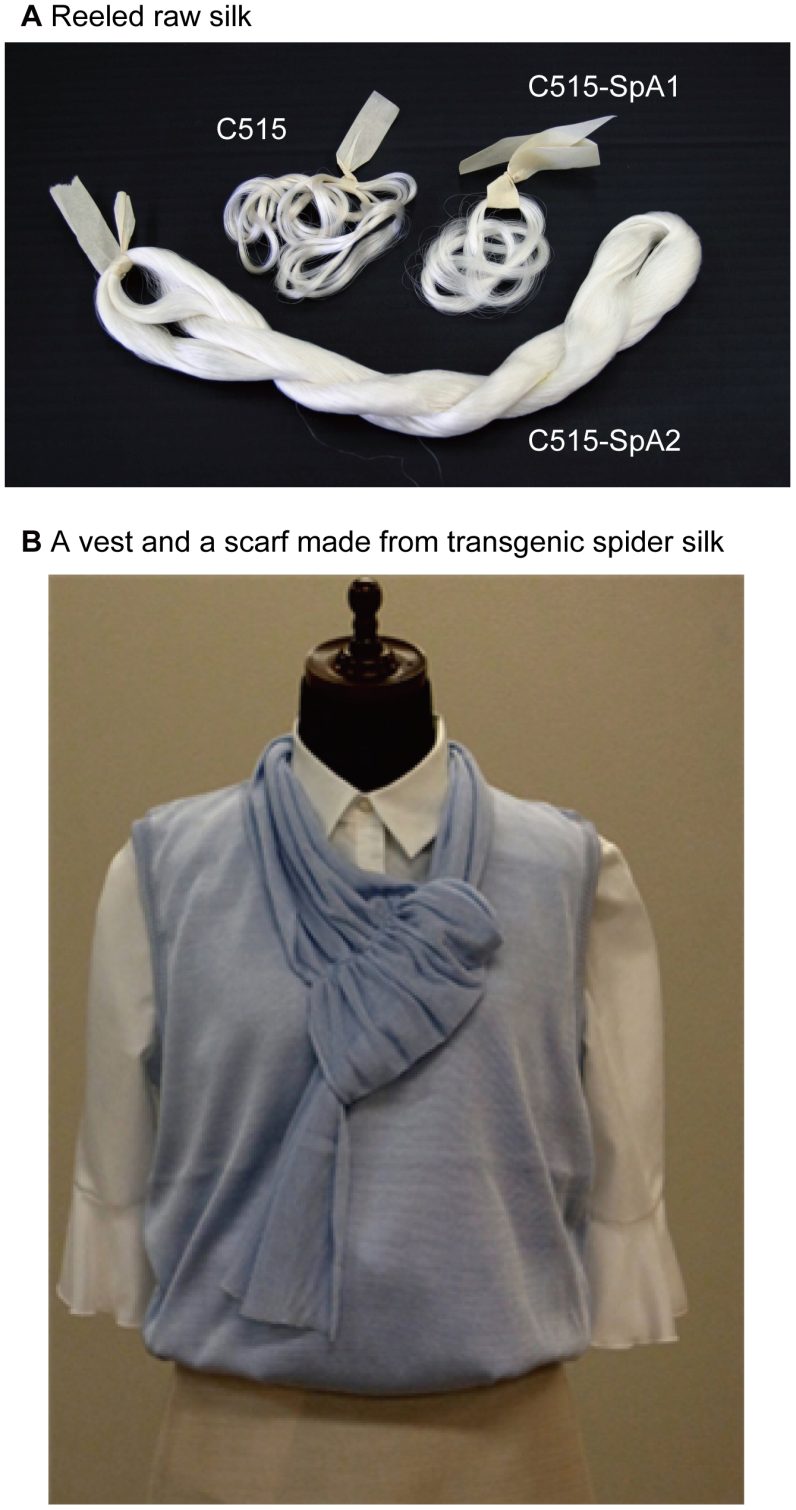
Raw silks and a woven vest and scarf knitted from transgenic spider silks. (A) Reeled raw silks of C515, C515-SpA1, and C515-SpA2 are shown. (B) A vest and scarf made by a knitting machine using C515-SpA2 to demonstrate the commercial possibilities of transgenic spider silk. Cocoons of C515-SpA2 were reeled by a reeling machine, woven, dyed, and knitted.

Because cocoons from transgenic silkworms produced in this study could be pulled into raw silk, mass production of raw silk was performed with the intent of machine knitting a vest and scarf. Figure 6 shows pictures of the raw silk yarn and the resultant woven vest and scarf, using C515-SpA2 silk. The weakest silk among the HC-SpA expressing transgenic silkworms was used for these articles of clothing. That it could be used with a knitting machine clearly demonstrates the strength and toughness of our transgenic silk for reeling. Moreover, the fabric was also dyed and sewn by a machine in an industrial setting. This demonstrates the use of the transgenic silks produced in this study as a strong, high-toughness raw silk in the textile industry.

## Conclusions

In this study, we cloned the spider (*Araneus ventricosus*) dragline protein gene, SpA, and generated a transgenic silkworm, which expressed the fusion protein of fibroin H-chain and SpA, HC-SpA, in cocoon silk. The raw silk prepared with transgenic cocoon-expressed spider-dragline silk protein showed improved breaking strain, breaking stress, and high toughness, nearly reaching that of spider dragline silk. We also demonstrated that resultant transgenic cocoons could be used directly in the textile industry.

Spider silk proteins can be used not only as fibers, but also as biomaterials for biomedical applications. In our group, silk fibroins have been used for regenerative medicine [31]–[34]. Silk containing the spider silk protein offers wide possibilities for biomedical applications, such as artificial tendons and ligaments, due to the ability to use transgenic technologies to control the tensile properties.

## Supporting Information

Figure S1 **Transfer plasmids for construction of transgenic silkworms.** Black boxes indicate the promoter and poly(A) signal region of the fibroin H-chain gene. Dark-grey boxes indicate the fibroin H-chain coding region. Bent lines indicate the intron sequences. White and light-grey boxes show SpA (A) and EGFP (B) ORF, respectively. Checked boxes indicate the 3xP3-DsRed2-poly(A) marker gene. Arrowheads show terminal repeats of the *piggyBac* transposon. The detailed construction of the transfer plasmid was described in a previous report [26]. After microinjection of these constructs into fertilized eggs with helper plasmid [25], the DNA region (surrounded by arrowheads) was introduced into the genome of the silkworm. The silkworms were then screened by DsRed expression in the eyes, and the silkworms C515-SpA1 and C515-SpA2 (using transfer plasmid (A)) and C515-EGFP (using transfer plasmid (B)), respectively, were established. The right-hand windows show the fusion proteins expressed in the posterior silk gland of the transgenic silkworms.
(TIF)

Figure S2 **Sample preparation.** (A) A single fiber from a cocoon was attached to a sampling sheet by adhesive tape. For tensile testing, each specimen was cut from the sheet and attached to a precise force meter. (B) To obtain the diameter of a single cocoon fiber, the diameter of a silk fiber was observed under a digital microscope and the value of “a” was determined. The cross-sectional area of a cocoon fiber was calculated assuming that its shape was elliptical, having a major axis “a” and a minor axis “b ( = a/2).”
(TIF)

Figure S3 **Sequence of the cloned Araneus ventricosus silk gene (SpA).** The cDNA sequence with its predicted translated amino acids is presented. The positions of the primers used for sequencing (arrows) and for second-strand cDNA amplification (bold letters) are indicated. A portion of the vector sequence (pBluescript II SK+, underlined) is indicated.
(TIF)

Figure S4 **Southern blotting analysis.** (A) The position of the probe was designed at the left arm of the *piggyBac* transposon. The digestion sites of *Kpn*I and *Hind*III are shown. (B) The genomic DNAs of parental individuals from each strain were analyzed. All genomic DNAs were digested with *Kpn*I or *Hind*III and subjected to Southern blotting analysis. All individuals had three independent transgene insertions.
(TIF)

Table S1 **Tensile properties of native and transgenic silk in cocoon fibers and raw silk.**
(DOC)

Table S2 **P-values from one-tailed t-tests of the cocoon fiber.**
(DOC)

Table S3 **P-values from one-tailed t-tests for raw silk.**
(DOC)
