# Supplementary figures and images for: High-Toughness Silk Produced by a Transgenic Silkworm Expressing Spider (Araneus ventricosus) Dragline Silk Protein
Source: PLoS One. 2014 Aug 27;9(8):e105325. doi: 10.1371/journal.pone.0105325 (PMC4146547; doi:10.1371/journal.pone.0105325)

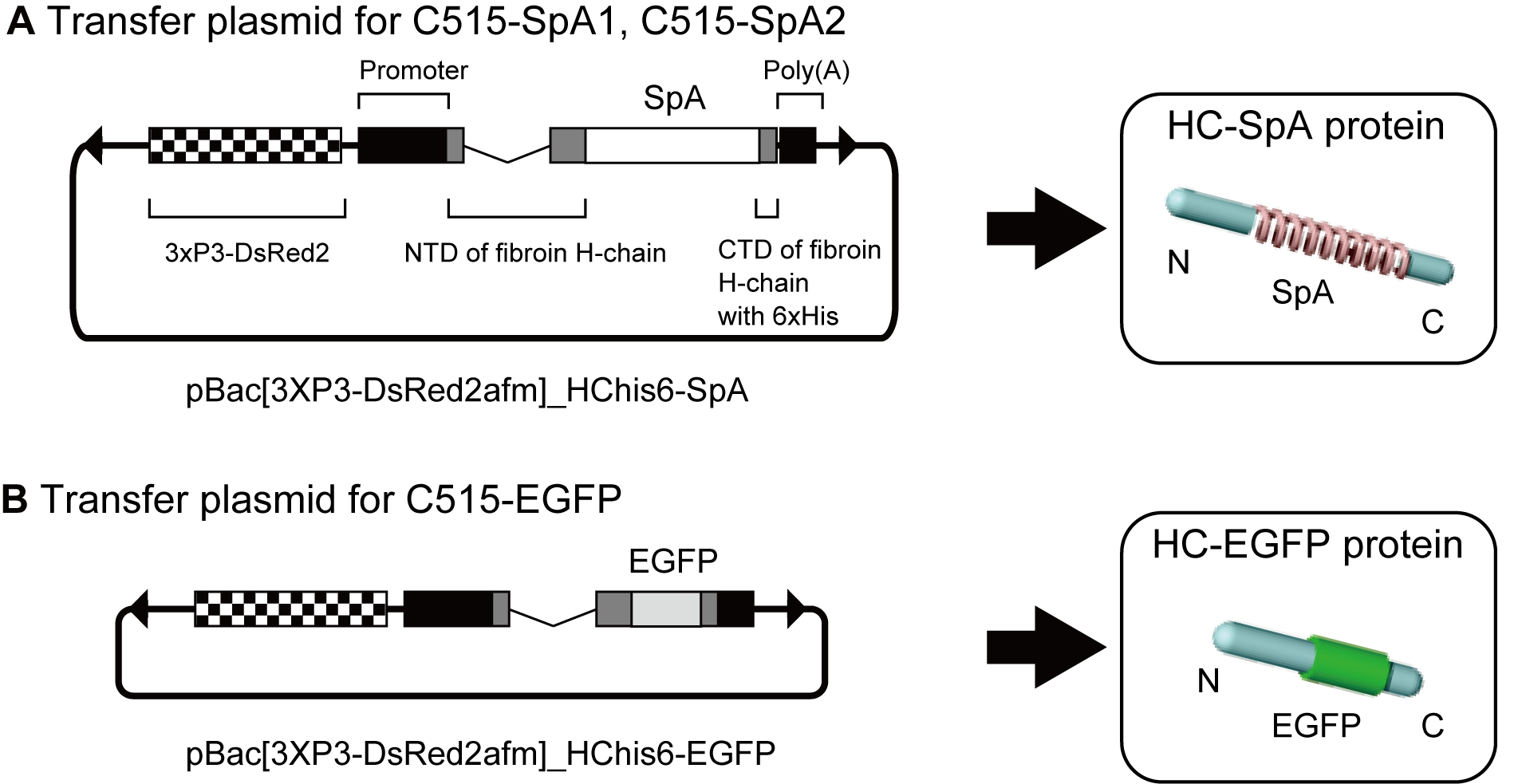

Supplement: Figure S1 — Transfer plasmids for construction of transgenic silkworms. Black boxes indicate the promoter and poly(A) signal region of the fibroin H-chain gene. Dark-grey boxes indicate the fibroin H-chain coding region. Bent lines indicate the intron sequences. White and light-grey boxes show SpA (A) and EGFP (B) ORF, respectively. Checked boxes indicate the 3xP3-DsRed2-poly(A) marker gene. Arrowheads show terminal repeats of the piggyBac transposon. The detailed construction of the transfer plasmid was described in a previous report [26]. After microinjection of these constructs into fertilized eggs with helper plasmid [25], the DNA region (surrounded by arrowheads) was introduced into the genome of the silkworm. The silkworms were then screened by DsRed expression in the eyes, and the silkworms C515-SpA1 and C515-SpA2 (using transfer plasmid (A)) and C515-EGFP (using transfer plasmid (B)), respectively, were established. The right-hand windows show the fusion proteins expressed in the posterior silk gland of the transgenic silkworms. (TIF) [file pone.0105325.s001.tif]

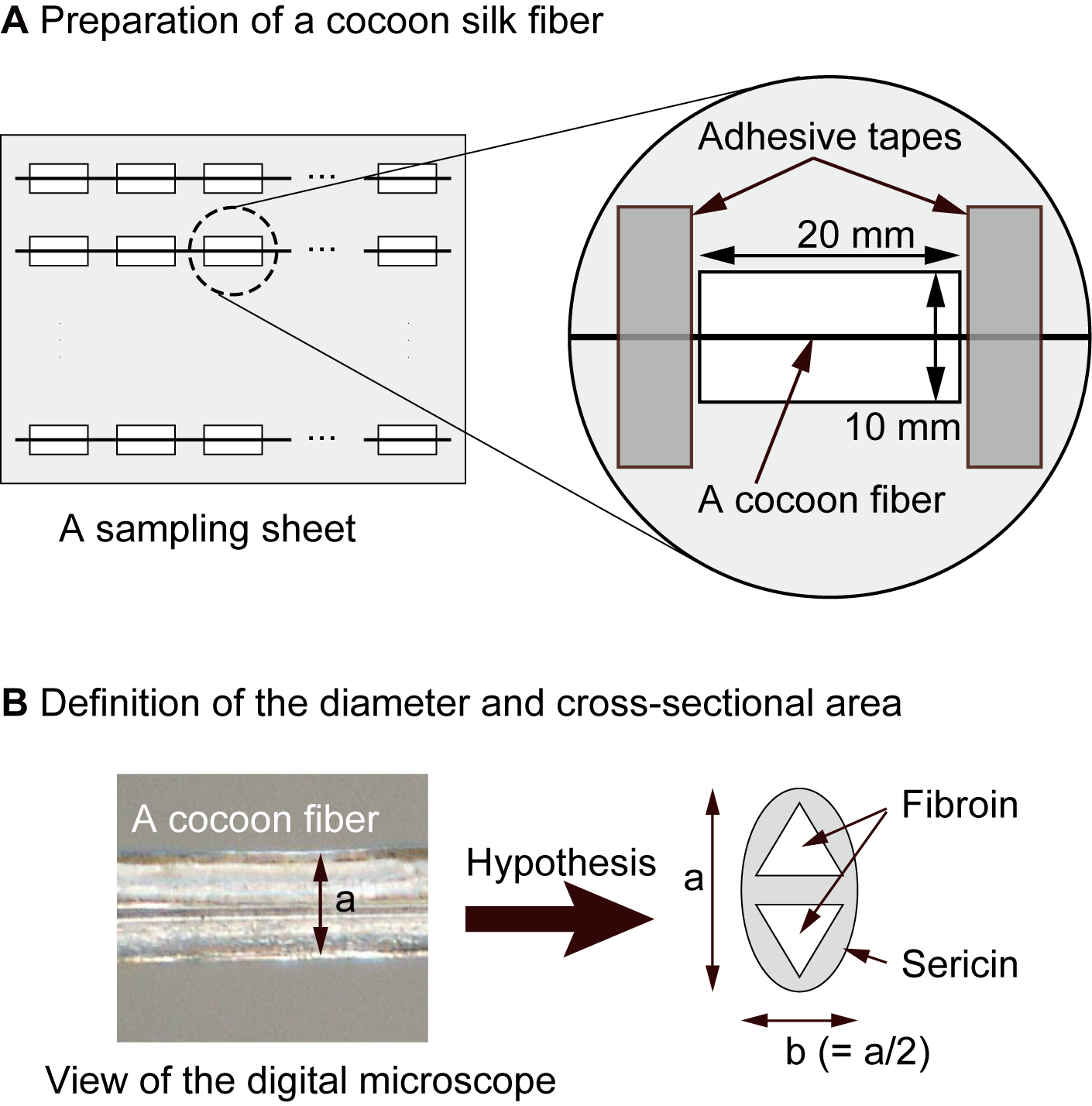

Supplement: Figure S2 — Sample preparation. (A) A single fiber from a cocoon was attached to a sampling sheet by adhesive tape. For tensile testing, each specimen was cut from the sheet and attached to a precise force meter. (B) To obtain the diameter of a single cocoon fiber, the diameter of a silk fiber was observed under a digital microscope and the value of “a” was determined. The cross-sectional area of a cocoon fiber was calculated assuming that its shape was elliptical, having a major axis “a” and a minor axis “b ( = a/2).” (TIF) [file pone.0105325.s002.tif]

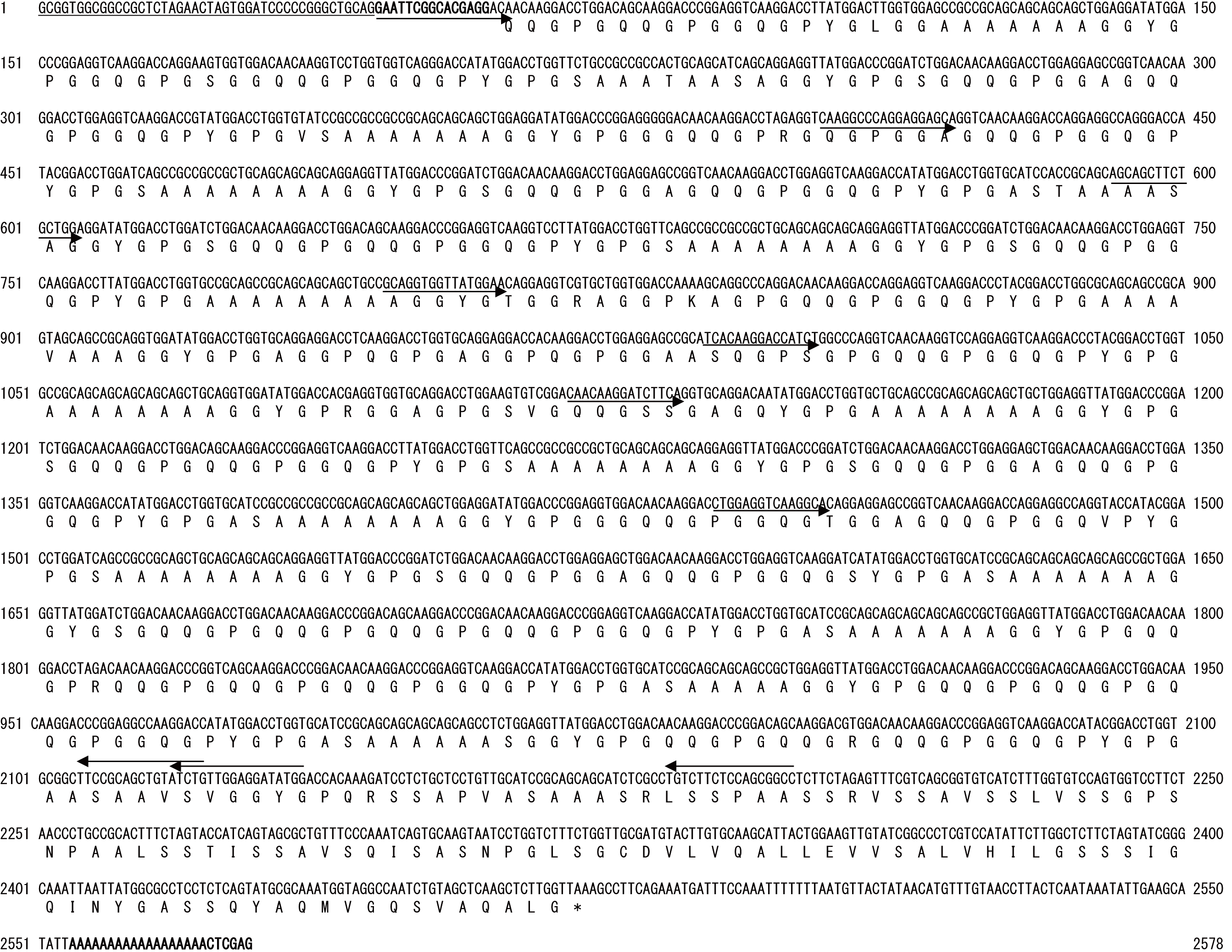

Supplement: Figure S3 — Sequence of the cloned Araneus ventricosus silk gene (SpA). The cDNA sequence with its predicted translated amino acids is presented. The positions of the primers used for sequencing (arrows) and for second-strand cDNA amplification (bold letters) are indicated. A portion of the vector sequence (pBluescript II SK+, underlined) is indicated. (TIF) [file pone.0105325.s003.tif]

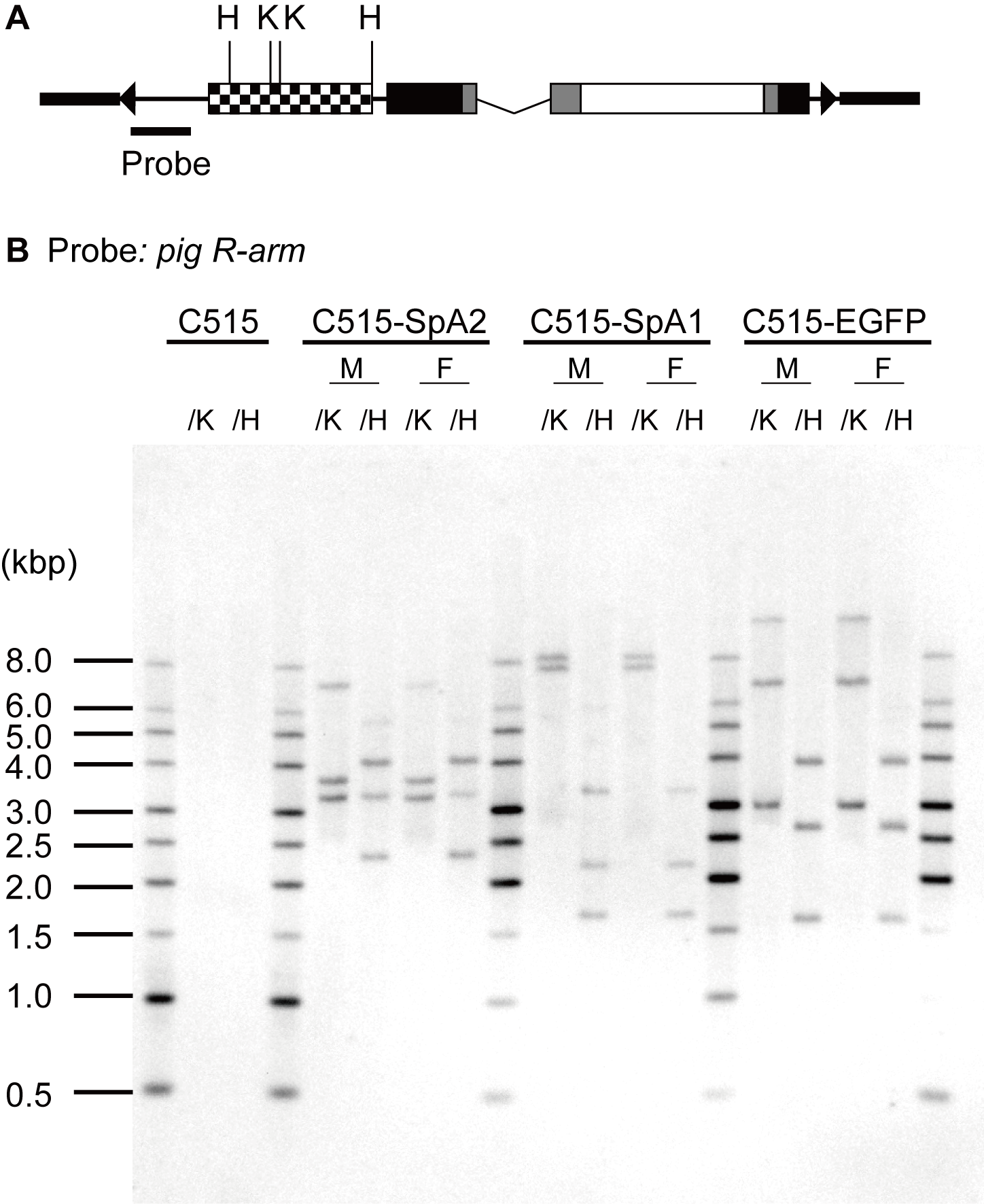

Supplement: Figure S4 — Southern blotting analysis. (A) The position of the probe was designed at the left arm of the piggyBac transposon. The digestion sites of KpnI and HindIII are shown. (B) The genomic DNAs of parental individuals from each strain were analyzed. All genomic DNAs were digested with KpnI or HindIII and subjected to Southern blotting analysis. All individuals had three independent transgene insertions. (TIF) [file pone.0105325.s004.tif]
