# Supplementary material for: High-Toughness Silk Produced by a Transgenic Silkworm Expressing Spider (Araneus ventricosus) Dragline Silk Protein
Source: PLoS One. 2014 Aug 27;9(8):e105325. doi: 10.1371/journal.pone.0105325 (PMC4146547; doi:10.1371/journal.pone.0105325)
Supplement: Table S1 — Tensile properties of native and transgenic silk in cocoon fibers and raw silk. (DOC) [file pone.0105325.s005.doc]

**Supporting Table 1. Tensile properties of native and transgenic silk in cocoon fibers and raw silk.**

1. Cocoon fibers

|  | **C515** (m=2, n=76) | | **C515-SpA1** (m=2, n=77) | | **C515-SpA2** (m=3, n=107) | | **C515-SpA1x2** (m=2, n=61) | | **C515-EGFP** (m=2, n=46) | |
| --- | --- | --- | --- | --- | --- | --- | --- | --- | --- | --- |
|  | Ave. | S.D. | Ave. | S.D. | Ave. | S.D. | Ave. | S.D. | Ave. | S.D. |
| Breaking Stress (MPa) | 293.7 | 38.1 | 334.4 | 23.4 | 318.1 | 71.3 | 359.9 | 47.1 | 263.6 | 40.6 |
| Breaking Strain (%) | 30.8 | 5.6 | 32.5 | 5.6 | 32.0 | 4.9 | 34.5 | 5.9 | 35.6 | 5.8 |
| Young’s modulus (MPa) | 5802.9 | 1043.2 | 6579.4 | 403.6 | 5923.6 | 812.7 | 7319.0 | 743.9 | 5295.6 | 972.9 |
| Toughness (MJ m^-3^) | 67.7 | 19.2 | 81.5 | 17.4 | 74.1 | 25.5 | 92.5 | 21.2 | 68.7 | 17.7 |

1. Raw silk

|  | **C515** (n=40) | | **C515-SpA1** (n=40) | | **C515-SpA2** (n=40) | | **C515-SpA1x2** (n=40) | | **C515-EGFP** (n=40) | |
| --- | --- | --- | --- | --- | --- | --- | --- | --- | --- | --- |
|  | Ave. | S.D. | Ave. | S.D. | Ave. | S.D. | Ave. | S.D. | Ave. | S.D. |
| Breaking Stress (MPa) | 521.3 | 50.5 | 575.0 | 16.3 | 574.7 | 18.6 | 591.7 | 35.0 | 487.7 | 13.7 |
| Breaking Strain (%) | 19.3 | 1.6 | 24.6 | 1.6 | 24.5 | 1.5 | 27.5 | 1.4 | 24.8 | 1.6 |
| Young’s modulus (MPa) | 13645.0 | 1238.8 | 14787.0 | 505.0 | 14664.6 | 610.9 | 14298.7 | 1058.3 | 13658.5 | 862.5 |
| Toughness (MJ m^-3^) | 75.8 | 11.6 | 104.2 | 8.6 | 103.0 | 8.6 | 116.1 | 9.5 | 91.9 | 7.1 |

(m: number of cocoons, n: number of specimens)
