# Supplementary material for: High-Toughness Silk Produced by a Transgenic Silkworm Expressing Spider (Araneus ventricosus) Dragline Silk Protein
Source: PLoS One. 2014 Aug 27;9(8):e105325. doi: 10.1371/journal.pone.0105325 (PMC4146547; doi:10.1371/journal.pone.0105325)
Supplement: Table S2 — P-values from one-tailed t-tests of the cocoon fiber. (DOC) [file pone.0105325.s006.doc]

**Supporting Table 2. *P*-values from one-tailed t-tests of the cocoon fiber.**

*P*-values were calculated using Student’s *t*-test (one-tailed) using Excel software.

1. Breaking stress

|  | **C515** | **C515-SpA1** | **C515-SpA2** | **C515-SpA1x2** |
| --- | --- | --- | --- | --- |
| **C515** | - | - | - | - |
| **C515-SpA1** | 0.00163 | - | - | - |
| **C515-SpA2** | 4.71E-13 | 0.0144 | - | - |
| **C515-SpA1x2** | 5.30E-16 | 4.91E-06 | 0.000110 | - |
| **C515-EGFP** | 3.39E-05 | 9.64E-09 | 2.79E-16 | 1.07E-19 |

1. Breaking strain

|  | **C515** | **C515-SpA1** | **C515-SpA2** | **C515-SpA1x2** |
| --- | --- | --- | --- | --- |
| **C515** | - | - | - | - |
| **C515-SpA1** | 0.0586 | - | - | - |
| **C515-SpA2** | 0.0314 | 0.269 | - | - |
| **C515-SpA1x2** | 0.000136 | 0.00197 | 0.0228 | - |
| **C515-EGFP** | 5.46E-06 | 4.63E-05 | 0.00168 | 0.151 |

1. Young’s modulus

|  | **C515** | **C515-SpA1** | **C515-SpA2** | **C515-SpA1x2** |
| --- | --- | --- | --- | --- |
| **C515** | - | - | - | - |
| **C515-SpA1** | 0.182 | - | - | - |
| **C515-SpA2** | 5.80E-09 | 1.54E-12 | - | - |
| **C515-SpA1x2** | 6.23E-20 | 4.57E-26 | 1.42E-12 | - |
| **C515-EGFP** | 0.00382 | 2.01E-05 | 4.03E-12 | 1.55E-25 |

1. Toughness

|  | **C515** | **C515-SpA1** | **C515-SpA2** | **C515-SpA1x2** |
| --- | --- | --- | --- | --- |
| **C515** | - | - | - | - |
| **C515-SpA1** | 0.0269 | - | - | - |
| **C515-SpA2** | 3.85E-06 | 0.0103 | - | - |
| **C515-SpA1x2** | 3.34E-11 | 2.30E-06 | 0.000557 | - |
| **C515-EGFP** | 0.391 | 0.0670 | 8.10E-05 | 9.64E-09 |
