# Supplementary material for: High-Toughness Silk Produced by a Transgenic Silkworm Expressing Spider (Araneus ventricosus) Dragline Silk Protein
Source: PLoS One. 2014 Aug 27;9(8):e105325. doi: 10.1371/journal.pone.0105325 (PMC4146547; doi:10.1371/journal.pone.0105325)
Supplement: Table S3 — P-values from one-tailed t-tests for raw silk. (DOC) [file pone.0105325.s007.doc]

**Supporting Table 3. *P*-values from one-tailed t-tests for raw silk.**

1. Breaking stress

|  | **C515** | **C515-SpA1** | **C515-SpA2** | **C515-SpA1x2** |
| --- | --- | --- | --- | --- |
| **C515** | - | - | - | - |
| **C515-SpA1** | 4.21E-08 | - | - | - |
| **C515-SpA2** | 3.22E-08 | 0.465 | - | - |
| **C515-SpA1x2** | 2.27E-10 | 0.00434 | 0.00423 | - |
| **C515-EGFP** | 9.99E-05 | 7.67E-38 | 2.039E-40 | 1.90E-23 |

1. Breaking strain

|  | **C515** | **C515-SpA1** | **C515-SpA2** | **C515-SpA1x2** |
| --- | --- | --- | --- | --- |
| **C515** | - | - | - | - |
| **C515-SpA1** | 2.67E-25 | - | - | - |
| **C515-SpA2** | 2.66E-25 | 0.400 | - | - |
| **C515-SpA1x2** | 1.09E-38 | 8.93E-14 | 5.99E-13 | - |
| **C515-EGFP** | 1.62E-26 | 0.213 | 0.298 | 4.36E-12 |

1. Young’s modulus

|  | **C515** | **C515-SpA1** | **C515-SpA2** | **C515-SpA1x2** |
| --- | --- | --- | --- | --- |
| **C515** | - | - | - | - |
| **C515-SpA1** | 9.45E-06 | - | - | - |
| **C515-SpA2** | 8.55E-07 | 0.166 | - | - |
| **C515-SpA1x2** | 0.00661 | 0.0314 | 0.00545 | - |
| **C515-EGFP** | 0.478 | 2.67E-08 | 5.69E-10 | 0.00200 |

1. Toughness

|  | **C515** | **C515-SpA1** | **C515-SpA2** | **C515-SpA1x2** |
| --- | --- | --- | --- | --- |
| **C515** | - | - | - | - |
| **C515-SpA1** | 1.65E-19 | - | - | - |
| **C515-SpA2** | 1.70E-20 | 0.254 | - | - |
| **C515-SpA1x2** | 3.26E-28 | 3.71E-09 | 5.87E-08 | - |
| **C515-EGFP** | 1.37E-10 | 7.53E-09 | 3.52E-10 | 2.12E-21 |
